# An Alabama-tailored human trafficking response protocol within the Community, Advocacy, Law, and Medicine framework: a state-adaptable model

**DOI:** 10.3389/fpsyt.2026.1885986

**Published:** 2026-08-13

**Authors:** Cassie Wicken, Luke Frazier, Marquita Brooks, Ellen Eaton, Hammond Lake, Kara S. Huls, Rebecca Rampe, Lindsey Baird Moultrie, Matthew Macaluso, Aniket Malhotra, Rachel Fargason, Li Li

**Affiliations:** 1 Department of Psychiatry and Behavioral Neurobiology, University of Alabama at Birmingham, Birmingham, AL, United States; 2 Love Immigration Law Firm, Birmingham, AL, United States; 3 Department of Psychology, University of Alabama at Birmingham, Birmingham, AL, United States; 4 Human Resources Communications, University of Alabama at Birmingham, Birmingham, AL, United States; 5 Department of Emergency Medicine, University of Alabama at Birmingham and Children’s of Alabama, Birmingham, AL, United States; 6 The University of Alabama at Birmingham Lister Hill Library, Birmingham, AL, United States

**Keywords:** human trafficking, immigrant health, labor trafficking, medical–legal partnership, multidisciplinary care, trauma-informed care, women’s health, healthcare protocol

## Abstract

Human trafficking is a major public health and human rights concern associated with high rates of psychiatric disorders, substance use disorders, chronic medical illness, and ongoing exposure to trauma. Patients frequently encounter healthcare systems while still experiencing exploitation, yet trafficking often goes unrecognized. Healthcare professionals are uniquely positioned to identify trafficking and facilitate access to recovery-oriented services. This manuscript describes the development and content of a trauma-informed healthcare response protocol designed to guide Alabama clinicians in identifying and supporting survivors of human trafficking. The protocol was developed through interdisciplinary collaboration among experts in psychiatry, psychology, emergency medicine, pediatrics, social work, and law, alongside engagement with community-based anti-trafficking organizations. It integrates national best-practice guidance with state-specific legal requirements and resource networks to support clinicians in responding to suspected trafficking in a trauma-informed and legally informed manner. Key components of the protocol include identification of clinical indicators of labor and sex trafficking, trauma-informed interviewing practices, structured screening and assessment approaches, safety planning strategies, and multidisciplinary referral pathways involving social work, psychiatric services, legal advocates, and community partners. The protocol also provides guidance regarding trauma-informed documentation practices, medical record safety considerations, and mandatory reporting requirements under Alabama law. Although developed within a specific legal and healthcare context, the protocol illustrates the importance of integrating local laws, institutional practices, and resource networks into trafficking-response frameworks. Its core principles of trauma-informed screening, multidisciplinary collaboration, and medical–legal coordination may be broadly adaptable across healthcare systems.

## 1 Introduction

Human trafficking represents a complex and underrecognized public health and human rights crisis associated with significant psychiatric morbidity, substance use disorders, and chronic medical conditions (1, 2). Survivors frequently experience major depression, post-traumatic stress disorder, anxiety disorders, and impaired social functioning, often in the context of exposure to coercion and violence (1, 3). These psychiatric sequelae are associated with repeated exposure to violence, psychological abuse, chronic stress, and pre-existing vulnerabilities, including prior trauma and social marginalization (1, 3, 4). These harms are influenced by broader structural conditions, including poverty, housing instability, migration vulnerability, and barriers to legal protection, that increase vulnerability to exploitation and complicate recovery (5–7).

While it is difficult to estimate the global prevalence of human trafficking, rising detection rates suggest that official statistics underreport the scope of the problem. The International Labour Organization has estimated that on a given day, approximately 50 million people are living under modern-day slavery, including people living in forced marriages (6). However, this estimate vastly exceeds the number of victims identified in official statistics. For example, the United Nations Office of Drugs and Crime reported 69,627 victims in 2022, utilizing a dataset of official statistics from 156 countries, comprising over 95 percent of the world’s population, and court case summary narratives from 113 countries (8). A substantial proportion of this gap between estimated trafficking and reported cases may stem from underreporting and the fact that human trafficking detection remains underdeveloped. Detection rates are trending upward, particularly detection rates with respect to labor trafficking. For example, there was a 25 percent increase in identified victims between 2019 and 2022 and a 47 percent increase in identified labor trafficking victims during the same period (8).

In the United States, the National Human Trafficking Hotline identified “10,359 potential trafficking situations involving 16,554 likely victims” in 2021; however, these figures likely underestimate the true prevalence of trafficking, particularly labor trafficking, due to significant barriers to identification, reporting, and data collection (9, 10).

Healthcare settings are structurally important sites for anti-trafficking intervention, as they may provide rare opportunities for private interaction between survivors and professionals, even in contexts where traffickers exert close control (11). A substantial proportion of trafficking survivors interact with healthcare systems during their exploitation (1, 9). For example, Polaris reported that in 2020 and 2021 respectively, 19 and 20 percent of victims whose interactions with “systems, institutions, or people” are known accessed health services during, upon escape from, or immediately after the exploitative situation. Only known interaction with family or friends (40 and 43 percent respectively) and interaction with law enforcement or the criminal justice system (23 percent in both years) were higher. A 2014 study of domestic sex trafficking survivors reported that of 98 survey participants who were trafficked and answered questions about their contact with healthcare, “87.8% had contact with a healthcare provider while they were being trafficked (12).” In this study, the most common treatment site, by far, was a “hospital/emergency room” (63.3 percent) (12).

Survivors may seek care for injuries, chronic illness, sexually transmitted infections, pregnancy, substance-related complications, or psychiatric symptoms related to trauma and coerced actions (11, 13). Yet these encounters frequently do not lead to recognition or intervention (11, 14). Trafficking frequently goes unrecognized across clinical and institutional settings due to limited training and awareness, systemic biases, stigmatization and mistrust surrounding mental health services, language barriers, fear of deportation among immigrant patients, and traffickers’ efforts to control communication and restrict help-seeking (4, 15). In addition, exploitative labor conditions may be normalized or misclassified as routine workplace hardship, further obscuring recognition (15). These failures indicate a persistent gap between understanding barriers to trafficking identification and implementing effective strategies to overcome them. This manuscript aims to help bridge this gap by blending practical knowledge gained in legal and medical settings with scholarship about overcoming these barriers.

Several healthcare-based initiatives have sought to improve identification and response to human trafficking through interdisciplinary, trauma-informed, locality-tailored models. For example, the THRIVE Clinic in Miami and the PATH Collaborative in Houston have demonstrated the feasibility of citywide, coordinated approaches that integrate medical, psychiatric, legal, and social services to support survivors (16). These models emphasize comprehensive needs assessment, care coordination, and the development of community partnerships to address the complex biopsychosocial needs of trafficking survivors. While prior models have demonstrated the feasibility of coordinated multidisciplinary responses, this manuscript focuses on translating those principles into a practical clinical protocol for healthcare providers operating within Alabama’s specific legal and service environment, integrating health, law, community, secure documentation, and local referrals. This state-specific focus emphasizes that trafficking-response protocols must be adapted to the legal, institutional, and community contexts in which they are implemented.

This manuscript describes the development of a structured clinical protocol designed to support healthcare providers in the identification, trauma−informed assessment, documentation, and multidisciplinary referral of patients experiencing human trafficking. The protocol was developed within the broader Community, Advocacy, Law, and Medicine (CALM) initiative at the University of Alabama at Birmingham, which integrates psychiatric care, legal advocacy, and community partnerships to guide healthcare providers in identifying and supporting survivors of human trafficking within clinical settings. The CALM model emphasizes a workflow that links identification, trauma-informed clinical assessment, and multidisciplinary intervention to downstream outcomes including legal stabilization, access to services, and recovery. The protocol integrates national best-practice guidance with state-specific legal requirements and referral pathways relevant to Alabama. The purpose of this manuscript is to describe the development, structure, and adaptation logic of the CALM protocol, to guide clinical responses to human trafficking in local contexts and to present the protocol as a practical model that may inform healthcare responses to trafficking in other regions. This protocol may serve as a template that can be revised for other states by substituting state-specific laws and resources.

## 2 Methods

### 2.1 Protocol development

The Community, Advocacy, Law, and Medicine (CALM) Healthcare Response Protocol for Identifying and Supporting Survivors of Human Trafficking in Alabama was developed through an interdisciplinary program-development process designed to translate existing research and national best-practice guidance into a practical clinical framework for healthcare providers. Development of the protocol was informed by three primary sources: a review of relevant literature, stakeholder engagement across sectors involved in trafficking response, and clinical experience with patients experiencing exploitation. The objective of the protocol was to operationalize trauma-informed trafficking response practices within healthcare workflows while integrating referral pathways to multidisciplinary services including medicine, social work, psychology, legal advocacy, and community organizations.

### 2.2 Literature review

A targeted review of the literature was conducted to identify existing research and guidance related to healthcare identification and response to human trafficking. Literature consideration and selection was informed by clinical observations, including repeated encounters with labor-trafficking survivors who reported prior interactions with healthcare systems during periods of exploitation without being identified. These observations prompted further exploration of barriers to recognition, available referral resources, and the broader service landscape for trafficking survivors. Stakeholder discussions and program-development needs arising during implementation of the CALM initiative also informed literature consideration and selection. Peer-reviewed literature and guidance from nationally recognized experts and organizations were prioritized for consideration, followed by scholarship and guidance produced by organizations and individuals with established experience in anti-trafficking research, survivor services, and program development. Sources included literature describing the health consequences of trafficking, studies examining identification of trafficking survivors in healthcare settings, and national guidance documents addressing trauma-informed responses to trafficking. Particular attention was given to literature addressing mental health sequelae of trafficking, labor trafficking dynamics, and interdisciplinary response models within healthcare systems. National guidance documents were also reviewed to identify recommended practices for trauma-informed screening, patient safety planning, and multidisciplinary coordination.

### 2.3 Stakeholder engagement

This protocol was informed by qualitative interviews of stakeholders involved in anti-trafficking response efforts across Alabama. The authors aimed to contribute to Alabama’s existing anti-trafficking ecosystem in a manner responsive to stakeholder capacities, priorities, and interests. Qualitative interviews were selected because they are well suited to understanding how stakeholders perceive needs, barriers, and opportunities within complex systems. As Patton observed, interviews are needed because “[w]e cannot observe how people have organized the world and the meanings they attach to what goes on in the world. (17, p. 628)”.

To begin finding participants, the authors utilized snowball sampling to find “information-rich key informants or critical cases” (17, p. 451). The authors held monthly virtual meetings, inviting anti-trafficking stakeholders from the community, interested healthcare providers and administrators, from the University of Alabama at Birmingham health system, and experts who have built anti-trafficking programs in other hospital-based settings.

Snowball sampling led to opportunity sampling and becoming participant-observers in Alabama’s larger anti-trafficking efforts (17). As Patton observed, “emergent flexible designs” are “one of the core strategic themes of qualitative inquiry” and “during fieldwork, the opportunity may arise to interview someone or observe an activity, neither of which could have been planned in advance” (17, p. 454). Members of the author team were invited to participate in Engage Together Alabama, a statewide anti-trafficking task force coordinated through the Alabama Attorney General’s Office that brings together representatives from healthcare systems, law enforcement, legal service providers, community organizations, and governmental agencies. Participation in task force activities and related discussions informed understanding of existing service gaps, referral pathways, and coordination challenges across systems responding to trafficking. For example, the authors learned that law enforcement in Alabama perceive disparate prosecution rates between sex and labor trafficking cases as a function of labor trafficking being difficult to prove due to lack of evidence and survivor unwillingness to come forward. Other opportunities which presented themselves included joint teaching opportunities with members of local community organizations and a medical-legal partnership with an immigration law firm designed to serve the mental health and legal needs of survivors. These partnerships facilitated discussions and revealed practical insights which informed development of practical clinical guidance and referral pathways appropriate to the local healthcare and legal context.

### 2.4 Clinical and programmatic experience

The protocol was further informed by clinical experience with the Community, Advocacy, Law, and Medicine (CALM) Clinic, a collaborative program based in an academic healthcare system providing psychiatric and multidisciplinary care to patients with histories of trafficking, exploitation, trauma, and related vulnerabilities and by one author’s experience consulting with and representing individuals seeking immigration-related legal services. Ethnographic observations from clinical encounters, along with ongoing collaboration with legal and community partners, helped identify common means of exploitation utilized by traffickers, clinical presentations, documentation challenges, and care coordination needs encountered when trafficking is suspected in healthcare settings. “Ethnographic inquiry takes as its central and guiding assumption that any human group of people interacting together for a period of time will evolve a culture”, and that culture informs how its participants see and interact with the world and what they deem possible (17, pp. 168-169). With this in mind, the authors sought to understand more about the culture of migrant labor. In serving the needs of immigrant populations, the authors observed that complex, precarious, and at times unlawful pathways facilitate access to work opportunities in the United States.

The authors observed that migration decisions were often informed by word-of-mouth information, kinship networks, and social relationships rather than a detailed understanding of formal immigration pathways. Employers often occupied positions that gave them access to information about immigration and employment systems that migrant workers did not possess, creating informational asymmetries that could be exploited. The authors observed that traffickers often exploited these informational asymmetries by threatening workers’ immigration status or employment opportunities. These insights guided the authors to search for formal research regarding these topics to inform the protocol.

CALM Clinic also benefited from regular virtual meetings that allowed stakeholders to share concerns and resources, providing a lens into the culture surrounding efforts to identify and assist trafficking survivors.

### 2.5 Relationship to the CALM model

Development of this protocol was closely aligned with the broader Community, Advocacy, Law, and Medicine (CALM) model developed in collaboration between academic psychiatry at the University of Alabama at Birmingham and the human trafficking practice group within Love Immigration Law Firm. The CALM model is an interdisciplinary framework designed to address human trafficking and related forms of exploitation through coordinated collaboration among healthcare providers, legal advocates, community organizations, and governmental partners. The model reflects the premise that effectively responding to trafficking survivors’ needs requires coordinated engagement across healthcare, legal, and community systems (4), particularly in contexts where systemic barriers limit access to care (7), and where legal protections and service access play a critical role in stabilizing survivors and facilitating recovery (18).

**Figure 1:**
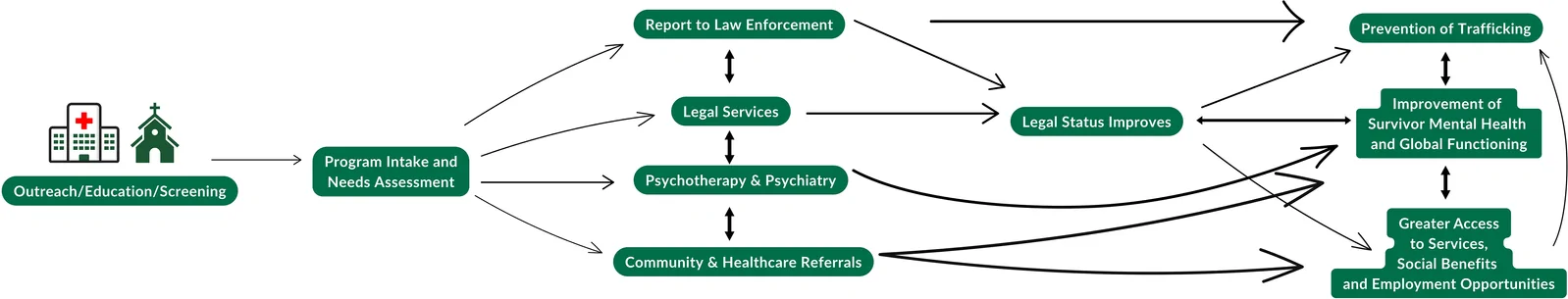
The Community, Advocacy, Law, and Medicine Framework for Multidisciplinary Collaboration.

**Figure 1** illustrates the operational structure of the CALM model, depicting the progression from outreach, screening, and intake through coordinated multidisciplinary interventions and the downstream impact on legal status, access to services, and survivor health and functioning.

The healthcare response protocol described in this manuscript was developed as a clinical component of this broader framework. In this context, the protocol functions as a practical tool for frontline healthcare providers to identify potential trafficking, initiate trauma-informed clinical responses, and facilitate referral to multidisciplinary services including psychiatric care, social work support, community advocacy, and legal consultation. However, the protocol was developed within a specific institutional and legal context, which may limit its direct application in other states or health systems without adaptation.

### 2.6 Ethical considerations

This project involved protocol development and program planning rather than human subjects research. No identifiable patient data were collected or analyzed for the purposes of this manuscript. Stakeholder engagement and consultations were conducted for program development and service planning purposes. Accordingly, institutional review board approval was not required.

## 3 Results: the CALM human trafficking response protocol

The protocol development process resulted in a structured clinical framework organized into the following domains: identification, screening, trauma-informed assessment, safety planning, multidisciplinary care coordination, and legal and documentation considerations. The framework also includes guidance for objective medical documentation of injuries, psychological symptoms, occupational hazards, and patterns of disrupted care, with particular attention to the identification of labor trafficking and documentation practices that protect patient safety.

### 3.1 Identification of patients experiencing trafficking

#### 3.1.1 Definition of human trafficking

Human trafficking is defined under U.S. federal law as the use of “force, fraud, or coercion” to compel labor or commercial sex acts (22 U.S.C § 7102(11)^1^). For minors involved in commercial sex, proof of force, fraud, or coercion is not required (22 U.S.C. § 7102(11)(A)^2^). Trafficking occurs across a wide range of industries and settings, including agriculture, construction, domestic work, hospitality, and the commercial sex trade. These definitions are established under U.S. federal trafficking statutes, including the Trafficking Victims Protection Act and federal sex−trafficking statute (22 U.S.C. § 7102^3^; 18 U.S.C. § 1591^4^), as well as Alabama state law (Ala. Code § 13A−6-152; 13A−6-153). Legal definitions relevant to healthcare identification are summarized in **Table 1**.

**Table 1:** Legal definitions of human trafficking under U.S. federal and Alabama law.

Situation	Counts as human trafficking when:
Commercial sex involving a minor	The person has not attained 18 years of age under federal law^a,b^ or is a ‘minor’ under Alabama law. Under Alabama law, ‘minor’ means a person under 19 years of age^c^.
Commercial sex involving an adult	“Force, fraud, or coercion” is used^a,b^.
Labor or services	Labor or services are obtained through “force, fraud, or coercion^b^.
Alabama statutory language	“Knowingly subjects another person to labor servitude or sexual servitude”^c^.

^a^Source: 18 U.S.C. § 1591 (2024); ^b^22 U.S.C. § 7102 (2024); ^c^Ala. Code § 13A−6 (2024).

Trafficking can occur within a variety of interpersonal and employment relationships. Data from the National Human Trafficking Hotline in 2021 indicate that the most common relationships between perpetrators and victims were employers (46%), family members (23%), and intimate partners (22%) (9). Although trafficking can occur in any population, certain vulnerabilities are frequently reported among survivors. Risk factors reported to the hotline in 2021 included recent migration or relocation (54%), mental or physical health concerns (10%), substance use concerns (9%), unstable housing (8%), runaway or homeless youth (7%), prior experiences of abuse or violence (6%), caretaker concerns such as caregiver substance use, neglect, or caregiver involvement in a sexualized industry (5%), child welfare system involvement or foster care (5%), gender or sexual minority status (4%), unaccompanied refugee minor status (3%), intellectual or developmental disability (3%), criminal history (3%), prior involvement in sexualized industries (2%), and economic hardship (1%) (9). Awareness of these vulnerabilities may assist clinicians in recognizing individuals who may be at increased risk of exploitation.

Data shows that females, gender minorities, and males are all at risk for sex and labor trafficking. However, among identified victims, sex trafficking disproportionately affects females, whereas identified labor-trafficking victims include a substantially larger proportion of males. This gender disparity appears far less pronounced for identified labor trafficking victims. Data from the National Human Trafficking Hotline in 2021 categorizing likely victims of trafficking according to gender (male, female, and gender minorities) and type (sex and labor) indicates that 48 percent of identified labor trafficking survivors were male, 22 percent were female, less than 1 percent were gender minorities, and 30 percent did not have a gender identified (9). Meanwhile, 79 percent of likely sex trafficking victims were female, 7 percent were male, 1 percent were gender minorities, and 13 percent did not have a gender identified. Likely sex trafficking victims (10,571 persons) greatly outnumbered likely labor trafficking victims (3,785) (9). According to the United Nations Office of Drugs and Crime, among known victims detected between 2020 and 2023 worldwide, 42 percent were trafficked for forced labor (8). 47 percent of these victims were men, 20 percent were boys, 23 percent were women, and 10 percent were girls (8). Meanwhile, 36 percent of victims were trafficked for sexual exploitation (8). 3 percent of these victims were men, 3 percent were boys, 64 percent were women, and 28 percent were girls (8).

These statistics likely understate the prevalence of labor trafficking and the trafficking of men. This is because labor trafficking survivors often lack awareness of their rights or are undocumented or otherwise fear deportation and because of the difficulties associated with investigating and prosecuting labor trafficking cases (10). Misclassification of labor trafficking as ordinary workplace hardship may contribute to the underidentification and underprosecution of labor trafficking, as well as the underidentification of male survivors (15). In addition, individuals experiencing labor trafficking may not conceptualize themselves as survivors of trafficking and may instead view their experiences as difficult but expected working conditions, further frustrating identification. This trend has been observed in the authors’ clinical practice regarding survivors identified while seeking legal advice regarding immigration status. Consistent with the broader literature, the authors encountered both male and female survivors of labor trafficking in clinical practice, while sex trafficking survivors were overwhelmingly female. Women and children were disproportionately represented among trafficked household members and dependents identified in these cases.

#### 3.1.2 Clinical indicators and risk factors

Identification of trafficking in healthcare settings can be challenging because patients often present for care without disclosing exploitation and may seek treatment for unrelated medical or psychiatric concerns (19). Traffickers may accompany patients, restrict communication, or coach responses, limiting disclosure and contributing to missed identification in clinical settings (11, 13, 19). Clinicians should therefore maintain a high index of suspicion when clinical presentations or social circumstances suggest possible coercion or exploitation (11, 19).

Patients may present with a range of medical, psychiatric, and social indicators. Clinical findings may include physical or occupational injuries, untreated or late-presenting acute or chronic conditions, sexually transmitted infections, substance use disorders, and symptoms of trauma and psychological distress (1, 2, 20). Patients may also present with non-specific somatic and psychological symptoms, including fatigue, gastrointestinal complaints, headaches, sleep disturbance, and trauma-related distress (3). Because these symptoms are common across many clinical populations, clinicians must consider patterns of behavioral, clinical, and contextual indicators rather than relying on a single diagnostic sign.

Behavioral cues may also suggest coercion or control (13). Patients experiencing trafficking may appear fearful or reluctant to speak in the presence of accompanying individuals, defer to another person when answering questions, demonstrate limited awareness of available avenues for seeking help, or provide incomplete or inconsistent histories (13, 19). These patterns may reflect coercive control, restricted access to information, and survivors’ limited recognition of their own exploitation (21, 22). Controlling companions may attempt to restrict direct communication with healthcare providers, including by speaking on behalf of the patient (13). Facilitating opportunities for private, independent communication, including the use of qualified interpreters rather than accompanying individuals, may help mitigate these barriers.

Occupational and environmental factors may also raise concern for trafficking. Individuals experiencing labor trafficking may present with injuries or untreated health conditions related to hazardous or unsafe working environments, including musculoskeletal strain, dehydration, and other consequences of neglect or abuse (10, 19). Individuals experiencing sex trafficking may present with injuries related to violence, sexually transmitted infections, pregnancy, and other reproductive health concerns (11, 19, 23).

Clinicians should avoid relying on gender-based assumptions when assessing possible trafficking. Although identified sex-trafficking victims are disproportionately female and identified labor-trafficking victims include a substantially larger proportion of males than identified sex-trafficking victims, trafficking occurs across all genders. Gendered expectations regarding who constitutes a “typical” trafficking survivor may contribute to underrecognition of male survivors, particularly in labor-trafficking settings, and may obscure exploitation experienced by women in labor sectors. Assessment should therefore focus on indicators of force, fraud, coercion, restricted autonomy, and exploitation rather than demographic characteristics alone.

A summary of potential clinical and contextual indicators of trafficking is provided in **Table 2**. The presence of one or more indicators does not confirm trafficking but should prompt clinicians to consider the possibility of exploitation and initiate trauma−informed assessment and screening.

**Table 2:** Clinical and contextual indicators of human trafficking in healthcare settings.

General red flags
• Behavioral: ○ Language barrier (24) ○ Accompanied by an individual who answers questions on their behalf or refuses privacy (25, 26) ○ Hesitant or fearful when responding to questions (29) ○ Not aware of time or date (26) ○ Unable to provide address or unaware of current location (29; 26) ○ Do not have control of identification documents (25, 26) ○ Inconsistent or scripted history (26, 29) • Social History: ○ Restricted movement; not free to come and go (26) ○ Behavior is subject to monitoring (24) ○ Works excessively long shifts (26) ○ Money is managed by someone else (26) ○ In debt to employer (26) • Medical: ○ Fatigue, gastrointestinal complaints, headaches, sleep disturbance, and trauma-related distress (3) ○ Musculoskeletal strain, dehydration, and other consequences of neglect or abuse (10, 19) ○ Exposure/illness due to unsanitary living conditions (23)
Labor Trafficking Indicators • Behavioral: ○ Shows fear or distrust towards supervisors (26) ○ Misrepresents job role (26) • Social History: ○ Living in the United States on a temporary visa or undocumented (24) ○ Threatened with deportation or imprisonment (24) ○ Employer falsely represented the terms or conditions of the job (24) ○ Required to reside at the workplace; cannot select their own housing (26) ○ Hazardous/unsafe/unsanitary workplace/living conditions (24) ○ Lives in overcrowded housing (26, 24) ○ Verbal and physical abuse at work (24) ○ Irregular work hours, lack of adequate breaks during work time, or no time off (26)
Sex Trafficking Indicators • Behavioral: ○ Controlled by a third party (such as a pimp, romantic partner, or spouse) (26) ○ Uses jargon specific to the sex trade (26) • Social History: ○ Employed in commercial sex venues (escort services, exotic dance, sex work, certain massage parlors) (26) ○ Runaway or homeless (23) ○ “History of sexual or physical abuse or neglect” (23) ○ “Interactions with Child Protective Services of Juvenile Justice System” (23) • Medical: ○ “Multiple sexual partners, unintended pregnancies, or sexually transmitted infections” (23) ○ Substance use (26) • Physical Appearance: ○ Tattoos or branding indicating ownership by another (26) ○ Inappropriate clothing for venue or weather (26)

Patients may experience barriers to communication and engagement with healthcare providers due to fear of retaliation, mistrust of institutions, language barriers, immigration concerns, and prior traumatic experiences (4). As Gomez et al. (27) observed, identifying trafficked patients may be “indirect and nuanced,” relying on trust-building, prior experiences, and documentation practices that can reveal patterns of concern across healthcare encounters over time.

### 3.2 Screening for human trafficking

Screening for human trafficking should be considered when clinical indicators or contextual risk factors raise concern for potential exploitation. Screening may be conducted by any trained clinical provider, including physicians, nurses, social workers, psychologists, and other members of the healthcare team.

Whenever possible, screening should occur in a private setting without accompanying individuals present. Clinicians should explain the purpose of screening questions and reinforce that participation in screening is voluntary (27). When language barriers exist, clinicians should facilitate confidential interpretation and culturally responsive communication to reduce barriers to care (27, 28). Clinicians should be mindful that multiple dialects may exist within the same language and that interpreters may not always share the patient’s dialect, potentially creating additional barriers to communication (27). Clinicians should ensure that patients and interpreters can clearly understand one another before relying on interpretation during clinical encounters. In practice, identifying an appropriate interpreter may require trial and error, particularly when multiple dialects exist within the same language. Even when communication is initially limited, interpreters may help clarify the patient’s specific dialect or linguistic background, allowing providers to more precisely communicate interpretation needs.

Structured screening tools may assist clinicians in identifying patients who may be experiencing trafficking. One such instrument is the Rapid Appraisal for Trafficking (RAFT), a brief validated screening tool validated for healthcare settings (14). The RAFT includes questions designed to identify experiences consistent with trafficking, including forced or deceptive labor, restrictions on leaving unsafe work conditions, threats or retaliation, and exchange of sex for basic needs such as shelter or food (14).

Clinicians should be aware that gendered assumptions may influence both screening practices and patient disclosure. Male survivors, particularly those experiencing labor trafficking, may be less likely to self-identify as trafficking survivors, while providers may be more likely to associate trafficking with sexual exploitation of women and girls. Screening should therefore be guided by indicators of coercion and exploitation rather than assumptions regarding gender, presentation, or type of exploitation.

In addition to validated screening tools, clinicians may use supplementary trauma−informed questions to explore experiences of coercion, exploitation, or control that may not be captured by structured instruments. Examples may include:

1. Coercion by intimate partners, family members, and other close relations:

“Sometimes people are pressured or threatened into doing things they do not want to do by a spouse, romantic partner, parent, guardian, or someone else close to them. Has anything like that ever happened to you?”.

2. Forced labor or financial control within family or household settings:

“Sometimes a person is made to work or earn income while someone else in the household controls how the money is used. Has anything like that ever happened to you? “.

3. Threats involving law enforcement, immigration authorities, and other authorities:

“Has anyone ever threatened to report you to law enforcement, immigration authorities, or another person or organization with power over you in order to make you do something against your wishes? “.

Positive responses to screening questions should prompt further trauma−informed assessment rather than being interpreted as definitive evidence of trafficking. Clinicians should prioritize assessment of the patient’s immediate safety and medical needs and readiness to engage with support services.

### 3.3 Trauma−informed interview and initial clinical response

When a patient screens positive for possible trafficking or when clinical concern for exploitation arises, providers should initiate a trauma−informed clinical interview. Trauma−informed care emphasizes patient safety, trust, autonomy, and collaboration while recognizing that survivors of trafficking may have experienced repeated trauma, coercion, and loss of control (14). Providers should avoid approaching clinical interviews as interrogations or attempts to confirm trafficking. The role of the healthcare provider is not to investigate criminal activity, but to assess medical needs, support patient safety, and facilitate access to appropriate services through a trauma-informed approach (3, 28).

Interviews should be conducted in a private setting whenever possible, as opportunities for disclosure may be compromised when others are present (27). These interactions should prioritize patient comfort, trust, and confidentiality (27). Clinicians should clearly explain their role, the purpose of questions being asked, and any applicable limits to confidentiality, including mandatory reporting requirements when applicable. Patients should be reminded that they may decline to answer questions or stop the conversation at any time. These practices are designed to help reduce fear and support patient autonomy, which may facilitate disclosure and engagement with care. Experiences of stigma, masculinity norms, fear of victimization labels, and concerns regarding credibility may influence disclosure differently across genders and should be considered during trauma-informed interviews.

During the clinical encounter, providers should assess the patient’s immediate medical and psychological needs while exploring potential indicators of coercion, exploitation, or restricted autonomy. Discussions should focus on the patient’s current safety, access to basic needs such as housing and food, and availability of supportive resources. Providers should avoid making assumptions about a patient’s circumstances and should respect the patient’s decisions regarding whether to disclose experiences of trafficking or engage with assistance.

### 3.4 Safety assessment and immediate patient needs

Survivors of trafficking may face ongoing threats from traffickers, unstable housing, food insecurity, untreated medical conditions, and limited access to supportive resources (19). A trauma-informed safety assessment should therefore evaluate immediate safety concerns, potential risk of retaliation or harm, housing stability, and available supportive resources (19).

Healthcare providers should also assess urgent medical and mental health needs. Survivors of trafficking experience high rates of depression, suicidal ideation, self-harm, post−traumatic stress disorder, substance use disorders, and other trauma−related conditions (3, 20). Accordingly, clinicians should consider screening for suicidal ideation and self−harm risk as part of the clinical evaluation when appropriate. Suicide risk assessment should follow standard clinical protocols, including evaluation of suicidal thoughts, prior suicide attempts, access to means, and protective factors.

Safety planning should be individualized and guided by the patient’s preferences and readiness to engage with services. Clinicians should avoid actions that may inadvertently increase risk to the patient’s safety or well-being, such as contacting law enforcement or external organizations without the patient’s consent unless legally required. Engagement with legal systems may carry unintended consequences for some patients, including immigration, criminal, or custody-related implications. When appropriate, referral to legal advocacy services may help patients better understand available options before additional disclosures or reports are made. When immediate safety concerns are identified, consultation with social work, behavioral health professionals, or institutional safety resources may help support development of an individualized, patient−centered safety plan (28).

Addressing immediate needs may also include treatment of acute medical conditions, management of injuries, access to food or clothing, and connection to safe housing or stabilization services. Coordination with multidisciplinary partners, including social workers, behavioral health providers, legal advocates, and community organizations, can facilitate appropriate referrals and continuity of care for patients experiencing trafficking (4). Safety concerns may vary across individuals and trafficking contexts, including threats related to intimate-partner violence, family relationships, employment, immigration status, housing, or child custody.

### 3.5 Multidisciplinary care coordination

Effective responses to human trafficking may require coordination across healthcare, legal, and community systems. Survivors often have complex medical, psychological, legal, and social needs that cannot be addressed by a single provider or institution (27, 28). Multidisciplinary collaboration can improve identification of trafficking and support patient safety (27, 28). In addition, it facilitates access to a range of services, including medical care, behavioral health treatment, legal advocacy, housing support, and case management (27, 28).

In Alabama, a range of organizations provide services for individuals experiencing or at risk for trafficking. To facilitate referral and coordination, clinicians may consult the Engage Together Alabama resource directory, a searchable statewide database of service providers supporting individuals affected by trafficking and related forms of exploitation (34). The directory includes healthcare providers, legal advocacy organizations, survivor service agencies, shelters, and community programs that offer support across the state. Access to such centralized resource networks can assist clinicians in identifying appropriate referral partners and coordinating multidisciplinary care.

This protocol is informed by the Community, Advocacy, Law, and Medicine (CALM) framework, which emphasizes collaboration among community organizations, advocacy services, legal professionals, and medical providers. Through coordinated partnerships, healthcare teams can connect patients experiencing trafficking with specialized services that address both immediate needs and longer−term recovery. Such collaboration may include referrals to social workers, behavioral health clinicians, community−based survivor service organizations, legal aid providers, and emergency or transitional housing programs, and supports continuity of care beyond the initial clinical encounter.

Healthcare providers should work closely with institutional partners such as social work departments, behavioral health services, and medical−legal partnerships when trafficking is suspected. Collaboration with community−based organizations may help address needs related to safe housing, legal protection, immigration support, employment assistance, and long−term recovery services.

#### 3.5.1 Care of patients with immigration concerns

Immigrant populations may be particularly vulnerable to labor and sex trafficking due to factors including language barriers, limited familiarity with legal systems, economic insecurity, and fear of immigration enforcement (4, 28). Traffickers frequently exploit immigration status as a mechanism of coercion, especially through threats of deportation and exploitation of visa systems in which a worker may lose legal immigration status if they leave their employer, but also through document confiscation and economic dependence (24). These dynamics may create substantial barriers to disclosure and help−seeking in healthcare settings (4, 13). Healthcare providers should approach patients who are immigrants using trauma−informed and culturally responsive practices (4). Providers should avoid making assumptions regarding immigration status.

Clinicians should be familiar with the interpreter services available within their institutions. For additional language support related to trafficking cases, providers may contact the National Human Trafficking Hotline at 1-888-373-7888, which may assist with access to trained interpreters and trafficking-specific resources (29).

Access to non−emergency services may vary depending on the clinical setting, insurance eligibility, and available safety−net resources. Referral to community health centers and other safety−net providers may therefore be important for ensuring continuity of care. Providers should be familiar with community organizations that specialize in supporting immigrant populations. In Alabama, the Hispanic and Immigrant Center of Alabama (HICA) provides legal services, community support, and advocacy for immigrants and may be an important referral partner for patients experiencing exploitation or legal vulnerability (35).

Some trafficking survivors may qualify for immigration protections designed for victims of trafficking and related crimes. These protections may include T nonimmigrant status (T visas) and Continued Presence (18). Early referral to qualified immigration attorneys or legal service organizations experienced in trafficking cases may therefore be an important component of comprehensive care for immigrant survivors.

#### 3.5.2 Crime victim compensation

Crime victim compensation may be a potential resource for patients who have experienced physical or psychological harm as a result of criminal conduct, including human trafficking, assault, abuse, or exploitation (33). In Alabama, the Crime Victims Compensation Commission may provide financial assistance for certain expenses related to the harm (35). Healthcare providers play a limited but important role in ensuring that patients are aware of this resource and are connected to appropriate support.

Providers should inform patients, in clear and simple language, that financial assistance may be available for individuals harmed by crime, but that eligibility for compensation programs requires reporting the crime and cooperating with law enforcement (35). Providers should then ask whether the patient would like assistance connecting with someone who can explain the program and help with the application. If the patient agrees, the provider should facilitate a referral, preferably through a warm hand−off, to a qualified legal services provider, survivor advocate, or social work team. A consultation with these professionals may allow patients to make an informed decision about whether pursuing compensation is in their best interest.

Information about eligibility requirements should be communicated in a neutral and non−coercive manner. Patients should not be pressured to report to law enforcement or to make decisions before they are ready. When appropriate, providers may note that earlier connection to services or documentation can sometimes support access to benefits, while also reassuring patients that delays do not necessarily preclude eligibility (35). Providers may inform patients experiencing trafficking that medical records may help support eligibility for compensation or other assistance programs.

Healthcare institutions are encouraged to establish and maintain referral relationships with legal services organizations, survivor advocacy programs, and internal social work or case management teams. These partnerships help ensure that patients receive accurate eligibility assessments and application support, while allowing healthcare providers to remain within their clinical role.

### 3.6 Legal and reporting considerations

Healthcare providers responding to suspected human trafficking must often navigate three interconnected responsibilities: fulfilling mandatory reporting obligations when legally required, respecting patient autonomy when reporting is discretionary, and documenting clinical findings in a manner that both protects patient safety and preserves medically relevant information. The following sections outline key considerations for clinicians in Alabama regarding reporting requirements, law−enforcement engagement, and documentation practices in cases involving suspected trafficking.

#### 3.6.1 Mandatory reporting requirements

Healthcare professionals in Alabama are mandated reporters of suspected abuse, meaning harm or threatened harm to a child’s health or welfare, and neglect (Ala. Code §§ 26-14-1^6^, 26-14-3(a)^7^). If a doctor, nurse, social worker, or mental health professional suspects that a minor may be a survivor of human trafficking, commercial sexual exploitation, or abuse or neglect more broadly, the provider must orally report this concern to the Alabama Department of Human Resources (DHR) immediately and follow up with a written report (Ala. Code § 26-14-3(a)^8^). Under Alabama law, mandatory reporting requirements regarding children apply when the patient is under 18 years old or is “under the age of 19 years … in need of protective services … and does not qualify for adult protective services under Chapter 9 of Title 38” (Ala. Code § 26-14-1(3)^9^). Given this nuance, clinicians should consider consulting their organization’s legal team when there is uncertainty regarding whether mandatory reporting obligations apply to an 18-year-old patient.

After a report is filed, DHR may initiate an investigation and coordinate with healthcare providers regarding the safety and disposition of the minor. Providers should explain the reporting requirement to the patient in a developmentally appropriate and trauma−informed manner. Although mandatory reporting limits confidentiality, clinicians should continue to involve the minor in care decisions whenever possible and provide emotional support throughout the process (28).

Alabama law establishes mandatory reporting obligations when healthcare providers and social workers have “reasonable cause to believe that a protected person has been subjected to abuse, neglect, exploitation, sexual abuse, or emotional abuse” (Ala. Code §§ 38-9-2^10^, 38-9-8^11^). Under Alabama law, a protected person generally includes individuals 18 years of age or older who, because of physical or mental impairment, neurodegenerative disease, intellectual or developmental disability, or other significant limitations, may be unable to adequately care for or protect themselves or their interests without serious consequences. In clinical practice, this may include patients whose condition substantially limits their ability to meet basic needs, protect themselves from harm, recognize or respond to exploitation, or make informed decisions regarding their safety. When mandatory reporting obligations apply, oral reports must be made immediately to the appropriate authorities, followed by a written report as required by law (Ala. Code § 38-9-8^12^).

Because the application of Alabama’s adult protective-services statutes involves case-specific legal and clinical judgment, clinicians should consider consulting their organization’s legal team when uncertainty exists regarding reporting obligations. As discussed above, even when mandatory reporting applies, providers should respect patient autonomy, seeking to involve patients in decision-making to the extent practicable under the circumstances.

#### 3.6.2 Law enforcement involvement, patient autonomy, and evidence preservation

Outside situations requiring mandatory reporting or when there is an immediate risk of harm, contact with law enforcement should not be automatic. In all other circumstances, patient autonomy should be paramount when considering whether to involve law enforcement, and clinicians should seek informed patient consent prior to engaging with law enforcement. Many trafficking survivors may be reluctant to engage with police due to fear of retaliation, immigration concerns, prior negative experiences with law enforcement, or ongoing safety concerns. When no mandatory reporting obligations apply and there is no immediate threat of harm, providers should respect a patient’s decision not to contact law enforcement.

When indicators of trafficking are present, clinicians should ask questions necessary to assess the patient’s immediate safety, medical needs, and appropriate referrals. Screening tools such as the Rapid Appraisal for Trafficking (RAFT) and related questions may assist clinicians in identifying possible exploitation. However, healthcare providers are not expected to conduct comprehensive investigative or forensic interviews regarding trafficking experiences. Detailed fact−finding is typically more appropriately conducted later by trained investigators, forensic interviewers, or legal professionals. The purpose of clinical questioning is therefore to obtain sufficient information to guide medical treatment, safety planning, and referral to appropriate services, while minimizing re−traumatization and avoiding duplication of later investigative interviews (25).

If a patient expresses interest in reporting trafficking, clinicians should first consider connecting the patient with an advocate or attorney experienced in working with trafficking survivors. Advocates and legal professionals can help patients understand potential risks and benefits of reporting, discuss available protections, and support patients in making informed decisions.

Healthcare systems should consider developing formal partnerships or memoranda of understanding (MOUs) with specialized law enforcement units trained in human trafficking and trauma-informed investigative practices. When such relationships are established, clinicians may contact designated officers if the patient requests law enforcement involvement or if urgent safety concerns arise. While involvement of an advocate or attorney remains the preferred approach when feasible, established MOU−based relationships may provide an intermediate level of safety and coordination compared with contacting law enforcement through standard channels. In the absence of these structured partnerships, clinicians should exercise caution when initiating direct law enforcement contact without patient consent.

Regardless of whether law enforcement becomes involved, clinicians should prioritize accurate documentation and preservation of potential evidence. Medical records may later play an important role in criminal investigations, civil litigation, or immigration proceedings. Documentation of injuries, occupational hazards, observed indicators of coercion, and patient statements should therefore occur even when a patient declines to involve police.

#### 3.6.3 Documentation, medical record safety, and legal remedies

Careful documentation is a critical component of the healthcare response to human trafficking. Providers should document clinical findings objectively and record patient statements using the patient’s own words whenever possible. Documentation may include physical injuries, occupational hazards, indicators of coercion or restricted autonomy, and the clinical reasoning supporting treatment recommendations or referrals. Clinicians should also document patterns that may reflect barriers to care associated with coercion or exploitation, such as missed appointments, delayed treatment for injuries or illness, interruptions in care, or situations in which patients report pressure to continue working despite medical advice. Documentation of referrals to mental health services, substance use treatment, infectious disease care, social services, or other long−term support can help establish the clinical basis for ongoing care needs (25, 30).

Detailed clinical documentation can play an important role in later criminal, civil, or immigration proceedings. However, the level of detail included in the medical record should remain guided by patient safety and the possibility that traffickers or controlling individuals could access the record. Practical strategies may include using confidential sections of the electronic health record, avoiding sensitive terminology in problem lists or after−visit summaries, and minimizing written materials that could place the patient at risk if discovered (31).

Recognition and careful documentation of trafficking−related health concerns may also facilitate access to legal protections and remedies for survivors. These may include immigration protections such as T nonimmigrant status (T visas) or Continued Presence, claims for unpaid wages or labor violations, civil litigation against traffickers, eligibility for victim compensation programs or civil settlement funds, vacatur or expungement of trafficking−related criminal records, protective orders, criminal investigation and prosecution of traffickers, and family law protections such as custody or divorce proceedings. Referral to attorneys or legal service organizations experienced in trafficking cases is recommended whenever possible to assist survivors in navigating these complex legal processes (18, 32).

## 4 Discussion

This protocol was developed to provide clinicians with a structured, trauma-informed framework for responding to suspected trafficking across healthcare settings. Rather than relying on *ad hoc* or purely discretionary responses, the framework integrates screening, safety assessment, documentation, multidisciplinary coordination, and referral processes into a cohesive clinical approach.

A key implication of this framework is the importance of embedding state-specific legal and resource context directly into clinical protocols. While the structure of identification and response may be generalizable, effective intervention depends on clinicians’ awareness of the legal and service environments in which they practice. This includes understanding mandatory reporting requirements, the scope of state-based survivor services, and the role of federal protections such as T visas. Variability in clinician familiarity with these legal and institutional frameworks may contribute to inconsistent responses across healthcare settings. By integrating this information into the protocol itself, providers are better equipped to make informed decisions about when and how to engage external systems, including social services, legal supports, and, when appropriate, law enforcement. In this way, the protocol not only guides clinical recognition but also clarifies potential pathways through which patients may access protection and support.

The protocol may be particularly relevant to psychiatric practice because trafficking frequently intersects with trauma-related disorders, suicidality, substance use, coercive control, institutional mistrust, and barriers to healthcare access. By providing a framework for recognizing indicators of exploitation, the protocol may help mental health professionals contextualize symptoms within the broader circumstances of coercion, trauma, and restricted autonomy. Recognition of trafficking may inform suicide-risk assessment by identifying sources of entrapment, fear, social isolation, and ongoing victimization that contribute to distress. Similarly, awareness of trafficking-related coercion may improve understanding of substance use patterns, particularly when substances are used as coping mechanisms, are introduced or encouraged by traffickers, or are intertwined with efforts to maintain control. The protocol may also help clinicians recognize how threats involving immigration status, employment, family relationships, housing, law enforcement, or other institutions can influence disclosure, treatment engagement, and help-seeking behavior. Finally, understanding the ways in which exploitation disrupts access to healthcare may assist clinicians in interpreting missed appointments, inconsistent treatment adherence, fragmented care histories, and other barriers to ongoing psychiatric treatment. While the protocol was not designed to evaluate psychiatric outcomes, it may support more comprehensive trauma-informed assessment, risk evaluation, and treatment planning for patients experiencing trafficking.

The protocol underscores the importance of accessible, up-to-date directories of local resources as a core component of trafficking response. Human trafficking initiatives can only coordinate to the extent that they are aware of one another, and in many settings, services remain fragmented or difficult to navigate. Models such as CALM can be strengthened by explicitly incorporating referral pathways and contact lists that reflect the local service landscape. In contexts where such infrastructure does not yet exist, engaging with or developing partnerships through regional coordinating bodies can help establish these connections.

Adaptation of this protocol to other jurisdictions will require consideration of local legal, institutional, and community contexts. Rather than assuming a single optimal model, healthcare systems may begin by mapping state-specific laws related to trafficking, mandatory reporting, child protection, adult protective services, and victim compensation. Development of multidisciplinary partnerships may be facilitated through engagement with existing anti-trafficking task forces, professional organizations, community-based service providers, and legal advocacy groups. Identification of local referral resources may benefit from stakeholder meetings and snowball-sampling approaches that help uncover organizations and services not readily visible through formal directories alone. Healthcare institutions should also consider developing clear reporting pathways, training clinical teams regarding relevant legal and community resources, and establishing relationships with attorneys, advocates, and community organizations. Future work should examine implementation strategies and evaluate how protocols function across diverse healthcare and legal environments.

The protocol also highlights the importance of avoiding gender-based assumptions in trafficking identification. Although identified sex-trafficking victims are disproportionately female and identified labor-trafficking victims include a larger proportion of males, trafficking affects individuals of all genders. Failure to recognize labor trafficking and male victimization may contribute to underidentification and missed opportunities for intervention. Future research should examine how gender influences disclosure, screening, referral patterns, and access to services.

### 4.1 Limitations and future directions

This protocol has not yet been formally evaluated in clinical practice, and its impact on trafficking identification, referral practices, and patient outcomes remains to be studied. Future research should examine implementation outcomes such as clinician adoption, screening rates, and referral patterns. This protocol offers a structured basis for future evaluation of feasibility, acceptability, adoption, and clinical impact. Because the protocol was developed within the legal and healthcare context of Alabama, adaptation may be necessary for use in other jurisdictions. Implementation will also require attention to local resource availability, institutional priorities, and the development of sustainable partnerships across sectors.

Protocol development was informed by a targeted rather than systematic review of the literature. Literature review and protocol development were guided by issues arising during clinical encounters and by concerns identified by medical, mental health, legal, social work, and governmental stakeholders. A more systematic review process may identify gaps, limitations, or areas for refinement.

Future work should also focus on implementation and evaluation of structured trafficking response protocols within healthcare systems. Important areas for development include clinician training, integration of screening and decision pathways into clinical workflows, and development of multidisciplinary partnerships that support referral and continuity of care. Additional study is needed to better understand how these protocols function in practice, including their impact on identification, referral patterns, provider confidence, and patient outcomes. Future studies may also help clarify how structured protocols influence coordination across healthcare, legal, and social-service systems. As healthcare systems continue to encounter trafficking survivors across clinical settings, ongoing refinement of practical response frameworks will remain important to supporting consistent, trauma-informed care.

## Data Availability

The original contributions presented in the study are included in the article/**Supplementary Material**. Further inquiries can be directed to the corresponding author.

## Footnotes

1 22 U.S.C. § 7102 (2024). *Definitions under the Trafficking Victims Protection Act*.

2 22 U.S.C. § 7102 (2024). *Definitions under the Trafficking Victims Protection Act*.

3 22 U.S.C. § 7102 (2024). *Definitions under the Trafficking Victims Protection Act*.

4 18 U.S.C. § 1591 (2024). *Sex trafficking of children or by force, fraud, or coercion*.

6 Ala. Code § 26-14-1 (2025). Code of Alabama, Title 26 – Infants and Incompetents, Chapter 14 – Reporting of Child Abuse or Neglect, Section 26-14-1 – Definitions.

7 Ala. Code § 26-14-3(a) (2025). Code of Alabama, Title 26 – Infants and Incompetents, Chapter 14 – Reporting of Child Abuse or Neglect, Section 26-14-3 – Mandatory Reporting.

8 Ala. Code § 26-14-3(a) (2025). Code of Alabama, Title 26 – Infants and Incompetents, Chapter 14 – Reporting of Child Abuse or Neglect, Section 26-14-3 – Mandatory Reporting.

9 Ala. Code § 26-14-1 (2025). Code of Alabama, Title 26 – Infants and Incompetents, Chapter 14 – Reporting of Child Abuse or Neglect, Section 26-14-1 – Definitions.

10 Ala. Code § 38-9-2 (2025). Code of Alabama, Title 38 – Public Welfare, Chapter 9 – Protection of Aged Adults or Adults with a Disability, Section 38-9-2 – Definitions.

11 Ala. Code § 38-9-8 (2025). Code of Alabama, Title 38 – Public Welfare, Chapter 9 – Protection of Aged Adults or Adults with a Disability, Section 38-9-8 – Reports by Physicians, Etc., of Physical, Sexual, or Emotional Abuse, Neglect, or Exploitation Required; Contents; Investigation.

12 Ala. Code § 38-9-8 (2025). Code of Alabama, Title 38 – Public Welfare, Chapter 9 – Protection of Aged Adults or Adults with a Disability, Section 38-9-8 – Reports by Physicians, Etc., of Physical, Sexual, or Emotional Abuse, Neglect, or Exploitation Required; Contents; Investigation.
