# Supplementary material for: An Alabama-tailored human trafficking response protocol within the Community, Advocacy, Law, and Medicine framework: a state-adaptable model
Source: Front Psychiatry. 2026 Aug 13;17:1885986. doi: 10.3389/fpsyt.2026.1885986 (PMC13519876; doi:10.3389/fpsyt.2026.1885986)
Supplement: Supplementary file 1 [file SupplementaryFile1.docx]

**Table 1. Legal definitions of human trafficking under U.S. federal and Alabama law**

| **Situation** | **Counts as Human Trafficking When:** |
| --- | --- |
| Commercial sex involving a minor | The person has not attained 18 years of age under federal law (18 U.S.C. § 1591(a)(2); 22 U.S.C. § 7102(11)(A)) or is a 'minor' under Alabama law.   Under Alabama law, 'minor' means a person under 19 years of age (Ala. Code § 13A-6-151). |
| Commercial sex involving an adult | “Force, fraud, or coercion” is used (18 U.S.C. § 1591(a)(2); 22 U.S.C. § 7102). |
| Labor or services | Labor or services are obtained through “force, fraud, or coercion” (18 U.S.C. § 1591; 22 U.S.C. § 7102(11)(B)). |
| Alabama statutory language | “Knowingly subjects another person to labor servitude or sexual servitude” (Ala. Code §§ 13A-6-152–153). |

*Sources: 18 U.S.C. § 1591 (2024); 22 U.S.C. § 7102 (2024); Ala. Code § 13A‑6 (2024)*

**Table 2. Clinical and contextual indicators of human trafficking in healthcare settings.**

**General Red Flags**

- Behavioral:
  - Language barrier (Polaris, 2019)
  - Accompanied by an individual who answers questions on their behalf or refuses privacy (Macias-Konstantopoulos, 2017; Macias-Konstantopoulos & Owens, 2018)
  - Hesitant or fearful when responding to questions (National Human Trafficking Resource Center)
  - Not aware of time or date (Macias-Konstantopoulos & Owens, 2018)
  - Unable to provide address or unaware of current location (National Human Trafficking Resource Center; Macias-Konstantopoulos & Owens, 2018)
- Do not have control of identification documents (Macias-Konstantopoulos, 2017; Macias-Konstantopoulos & Owens, 2018)
  - Inconsistent or scripted history (National Human Trafficking Resource Center; Macias-Konstantopoulos & Owens, 2018)
- Social History:
  - Restricted movement; not free to come and go (Macias-Konstantopoulos & Owens, 2018)
  - Behavior is subject to monitoring (Polaris, 2019)
  - Works excessively long shifts (Macias-Konstantopoulos & Owens, 2018)
  - Money is managed by someone else (Macias-Konstantopoulos & Owens, 2018)
  - In debt to employer (Macias-Konstantopoulos & Owens, 2018)
- Medical:
  - Fatigue, gastrointestinal complaints, headaches, sleep disturbance, and trauma-related distress (Hopper & Gonzalez, 2018)
  - Musculoskeletal strain, dehydration, and other consequences of neglect or abuse (Eyerman et al., 2023; Gordon et al., 2018)
  - Exposure/illness due to unsanitary living conditions (Tracy & Macias-Konstantopoulos, 2017)

**Labor Trafficking Indicators**

- Behavioral:
  - Shows fear or distrust towards supervisors (Macias-Konstantopoulos & Owens, 2018)
  - Misrepresents job role (Macias-Konstantopoulos & Owens, 2018)
- Social History:
  - Living in the United States on a temporary visa or undocumented (Polaris, 2019)
  - Threatened with deportation or imprisonment (Polaris, 2019)
  - Employer falsely represented the terms or conditions of the job (Polaris, 2019)
  - Required to reside at the workplace; cannot select their own housing (Macias-Konstantopoulos & Owens, 2018)
  - Hazardous/unsafe/unsanitary workplace/living conditions (Polaris, 2019)
  - Lives in overcrowded housing (Macias-Konstantopoulos & Owens, 2018; Polaris, 2019)
  - Verbal and physical abuse at work (Polaris, 2019)
  - Irregular work hours, lack of adequate breaks during work time, or no time off (Macias-Konstantopoulos & Owens, 2018)

**Sex Trafficking Indicators**

- Behavioral:
  - Controlled by a third party (such as a pimp, romantic partner, or spouse) (Macias-Konstantopoulos & Owens, 2018)
  - Uses jargon specific to the sex trade (Macias-Konstantopoulos & Owens, 2018)
- Social History:
  - Employed in commercial sex venues (escort services, exotic dance, sex work, certain massage parlors) (Macias-Konstantopoulos & Owens, 2018)
  - Runaway or homeless (Tracy & Macias-Konstantopoulos, 2017)
  - “History of sexual or physical abuse or neglect” (Tracy & Macias-Konstantopoulos, 2017)
  - “Interactions with Child Protective Services of Juvenile Justice System” (Tracy & Macias-Konstantopoulos, 2017)
- Medical:
  - “Multiple sexual partners, unintended pregnancies, or sexually transmitted infections” (Tracy & Macias-Konstantopoulos, 2017)
  - Substance use (Macias-Konstantopoulos & Owens, 2018)
- Physical Appearance:
  - Tattoos or branding indicating ownership by another (Macias-Konstantopoulos & Owens, 2018)
  - Inappropriate clothing for venue or weather (Macias-Konstantopoulos & Owens, 2018)
